# TREM2 expression in the human brain: a marker of monocyte recruitment?

**DOI:** 10.1111/bpa.12564

**Published:** 2017-10-30

**Authors:** Marie Fahrenhold, Sonja Rakic, John Classey, Carol Brayne, Paul G. Ince, James A. R. Nicoll, Delphine Boche, MRC‐CFAS

**Affiliations:** ^1^ Clinical Neurosciences, Clinical and Experimental Sciences Academic Unit Faculty of Medicine, University of Southampton Southampton SO16 6YD UK; ^2^ Cambridge Institute of Public Health, Department of Public Health and Primary Care University of Cambridge Cambridge CB1 8RN UK; ^3^ Sheffield Institute for Translational Neuroscience Sheffield University Sheffield S10 2HQ UK; ^4^ Department of Cellular Pathology University Hospital Southampton NHS Foundation Trust Southampton Southampton SO16 6YD UK

**Keywords:** TREM2, human brain, dementia, microglia, monocyte, neuropathology

## Abstract

Mutation in the triggering receptor expressed on myeloid cells *(TREM) 2* gene has been identified as a risk factor for several neurodegenerative diseases including Alzheimer's disease (AD). Experimental studies using animal models of AD have highlighted a number of functions associated with TREM2 and its expression by microglial cells. It has therefore been assumed that this is also the case in humans. However, there is very limited information concerning the cellular expression of TREM2 in the human brain. As part of investigations of microglia using *post‐mortem* resources provided by the Medical Research Council Cognitive Function and Ageing Studies (MRC‐CFAS), we immunostained the cerebral cortex of 299 participants for TREM2 using the Sigma antibody HPA010917 and compared with the macrophage/microglial markers Iba1 and CD68. As expected, Iba1 and CD68 labeled microglia and perivascular macrophages. However, in most cases (284/299), the TREM2 antibody labelled monocytes within vascular lumens, but not microglia or perivascular macrophages. In contrast, in 5 out of 6 cases with acute infarcts, TREM2 immunoreaction identified cells within the brain parenchyma interpreted as recruited monocytes. Six cases with old infarcts contained phagocytic foamy macrophages which were CD68‐positive but TREM2 negative. Our observations, using the HPA010917 anti‐TREM2 antibody, suggest that TREM2 is not expressed by microglia but instead seems to be a marker of recruited monocytes in the human brain. This finding has implications with regards to the role of TREM2 as a risk factor, emphasizing the importance of systemic immune responses in the development and progression of Alzheimer's disease.

## Introduction

Heterozygous mutation in triggering receptor expressed on myeloid cells *(TREM) 2* gene has been identified as a risk factor for several neurodegenerative diseases including Alzheimer's disease (AD) 10, 21, amyotrophic lateral sclerosis 5, and Parkinson's disease 25. TREM2 is a transmembrane receptor of the immunoglobulin family, which associates with the adapter protein DAP12 for signaling. The TREM2/DAP12 pathway is involved in the activation of human dendritic cells derived *in vitro* from monocytes 2 and thus TREM2 is thought to be involved in innate immunity. The importance of *TREM2* in brain function has been illustrated by the autosomal recessive disorder Nasu‐Hakola disease (NHD) due to homozygous loss‐of‐function mutations in the TREM2 gene 11, 32, in which affected people develop a presenile frontotemporal dementia with sclerosing leukoencephalopathy and polycystic lipomembranous osteodysplasia. Neuropathological features of NHD include demyelination and massive gliosis 42. It was then hypothesized that microglia, as the resident immune cells of the brain, might express TREM2. This was confirmed in adult murine microglia with regional variations of TREM2 expression in the brain 12, 36, and it was suggested that TREM2 was implicated in the phagocytosis of apoptotic neurons without triggering an inflammatory response 17, 38, an essential function of healthy microglia.

*TREM2* mutation was identified as a significant risk factor for AD with an effect size comparable with that of the ɛ4 allele of apolipoprotein E (*APOE*) gene 10, 21, although it is considerably less common. The role of TREM2 in the brain has been mainly investigated in different mouse models of AD. In APP23 transgenic mice, which carry the human Swedish mutation *APP* KM670/671NL, TREM2 expression was observed to be associated with the progression of amyloid deposition with microglia clustering around amyloid plaques 8. While in the *APP/PS1* transgenic mice (Swedish and PSEN1 L166P mutations) lacking one copy of the *TREM2* gene, a reduced number of microglia associated with plaques was observed without alteration of the amyloid load 41. Recently it was shown that in the 5XFAD transgenic mice (5 point mutations in the human *APP* and *PSEN1* genes), the function of microglial TREM2 is to sense lipids promoting microglial survival and clustering around Aβ plaques 43, supporting a protein microarray study that identified the apolipoproteins ApoE and Clusterin/ApoJ as ligands for human TREM2 44. Despite the discrepancies in these studies regarding the role of TREM2 in amyloid deposition, possibly due to the differences in the transgenic mouse models used, the findings are consistent with a direct or indirect role for TREM2 in myeloid cell migration, proliferation, survival and phagocytosis.

In contrast to animal studies, investigation to localize TREM2 expression in the human brain has been sparse. In a *post‐mortem* study involving 11 controls, 11 possible AD and 11 AD cases, the highest level of TREM2 by Western blot was found in the AD cases followed by the possible AD cases compared to controls, using the R&D antibody AF1828. By double staining with this antibody and the microglial marker HLA‐DR, they observed TREM2 expressed in some microglia and neurons 28. A survey of 7 commercial TREM2 antibodies on formalin‐fixed paraffin‐embedded *post‐mortem* brain tissue found only 3 antibodies to bind with human recombinant TREM2 protein on Western blot, performed to test their specificity. The two best TREM2 antibodies were the Sigma HPA010917 and the R&D AF1828 antibodies 34, also validated using the same methodology by the companies Sigma (www.proteinatlas.org) and R&D. Therefore, to investigate the localization of TREM2 in the human brain, we have used the cohort of the Medical Research Council Cognitive Function and Ageing Study (MRC CFAS) in which we have also studied multiple other microglial proteins 31. We screened 299 brains unselected from the whole spectrum of cognitive function found in population samples, including any dementia type and treatment but characterized in terms of clinical and neuropathological data 3, 35 with the HPA010917 and AF1828 anti‐TREM2 antibodies.

## Materials and Methods

### The CFAS cohort

The Medical Research Council Cognitive Function and Ageing Study (MRC CFAS) has been recruiting individuals living in the community aged 65 years and over since 1990 3. The main aims were to estimate the prevalence and incidence of cognitive decline and dementia; to determine the rate of progression of cognitive decline and survival, and to identify risk factors for cognitive decline and dementia. Participants were invited to consent to brain donation after death. The ascertainment of dementia status at death has been described in detail 35 and was based on review of information available from death certificates, last interview assessment and the informants' information about participants' function and cognition (Mini Mental State Examination – MMSE‐ score) during the last years of life. Brains sections from 299 participants were used in this study with the demographic and cognitive profile of the cohort described in Table 1. All analyses were conducted blind to *in vivo* state.

**Table 1:** Characteristics of the cohort according to dementia status.

	No dementia (*n* = 130)	Dementia non‐AD pathology (*n* = 65)	Dementia with AD pathology (*n* = 83)	Unknown dementia status (*n* = 21)
Number of women^*^	66 (51)	49 (75)	53 (64)	10 (48)
Age at death (years)^†^	84 (77; 90)	89 (85; 93)	89 (83; 93)	86 (84; 91)
Years since last cognitive assessment^†^	1·1 (0·5; 1·8)	1·7 (0·8; 3·0)	1·5 (0·8; 3·2)	2·5 (2·0; 3·4)
MMSE at last assessment^†^	25 (22; 28)	18 (11; 23)	11 (6; 17)	25 (22; 27)

*n(%).

^†^Median (interquartile range).

AD = Alzheimer's disease; MMSE = mini‐mental state examination.

### Immunohistochemistry

**Figure 1:**
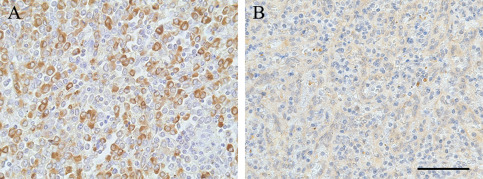
*TREM2 immunostaining in spleen with (A) the anti‐TREM2 antibody HPA010917 from Sigma and with (B) the anti‐TREM2 AF1828 from R&D*. Hematoxylin counterstaining, scale bar = 50 μm.

Optimization of the staining for the polyclonal rabbit anti‐TREM2 antibody HPA010917 (Sigma, Gillingham, UK) and the polyclonal goat anti‐TREM2 AF1828 (R&D, Abingdon, UK) was performed using different antigen retrieval pretreatments on splenic tissue, a myeloid organ used as a positive control. These included: (i) no pretreatment, (ii) heat retrieval pretreatment with either (a) citrate buffer pH6 or (b) EDTA buffer pH8. The best immunodetection was obtained with the HPA010917 anti‐TREM2 antibody at a concentration of 1:100 after EDTA pH8 microwave pretreatment (Figure 1).

Four micrometer sections of formalin‐fixed paraffin‐embedded tissue from the middle frontal gyrus were screened for TREM2 protein immunoreactivity using the HPA010917 anti‐TREM2 antibody under the same conditions.

Biotinylated secondary antibody from Dako (Glostrup, Denmark) was visualized using the avidin‐biotin‐peroxidase complex method (Vectastain Elite ABC from Vector Laboratories (Peterborough, UK)) with 3,3′ diaminobenzidine (DAB, Vector Laboratories (Peterborough, UK)) as chromogen and 0.05% hydrogen peroxide as substrate. All sections were counterstained with hematoxylin then dehydrated before mounting in DePeX (VWR International, Lutterworth, UK). For each run, sections incubated in the absence of the primary antibody were included as negative controls, and a section of spleen was included as a positive control to ensure staining consistency across the different runs (Figure 1A).

All 299 cases were assessed for parenchymal TREM2 immunostaining blinded to the identity of the cases by 2 independent assessors (MF and DB). Morphological pathological features were assessed on sections stained with hematoxylin and eosin (H&E). The identification and assessment of the infarcts was performed by an experienced neuropathologist (JARN) on the H&E stained slides according to the current classification (Table 2) 7, 16, 22. TREM2‐positive cells were scored within the identified infarcts using x20 objective magnification and reported as: – = 0; + = 1–20; ++ = 21–50; +++ = >51 per field; and for CD68‐positive cells as: – = 0; + =1–20; ++ = 21–100; +++ = >101 per field.

**Table 2:** Morphological characterization of lesion age 7, 16, 22.

Very acute infarct	<24 h	Neuronal ischemia, very early neuropil fragmentation, no macrophages, no neutrophils, no microvascular proliferation, no astrocytosis
Acute infarct	1–3 days	Neuronal ischemia, early neuropil degeneration, no macrophages, ±neutrophils, no microvascular proliferation, no astrocytosis
Subacute infarct	approx. 1 week	Neuronal ischemia, neuropil degeneration, foamy macrophages, no neutrophils, microvascular proliferation, surrounding early astrocytosis
Old infarct	several weeks to months/years	Neuronal loss, advanced neuropil degeneration (cavitated), +/−foamy macrophages, no neutrophils, no microvascular proliferation, surrounding astrocytosis

Sections previously stained for the microglial phagocytic markers Iba1 (rabbit polyclonal, Wako, Japan) and CD68 (clone PG‐M1, Dako Glostrup, Denmark) 31 were reviewed.

## Results

### TREM2 expression in the spleen

The spleen contains numerous monocytes/macrophages and so is a suitable choice for a TREM2 positive control for immunohistochemistry. Only the Sigma antibody HPA010917 was able to detect the monocytes/macrophages (Figure 1A). No staining was obtained on the same spleen with the R&D AF1828 antibody despite the use of different pretreatments for antigen retrieval (Figure 1B). Therefore, the CFAS brain sections were screened for TREM2 expression using the HPA010917 antibody.

### Microglia and perivascular macrophages in the human brain are TREM2 negative

**Figure 2:**
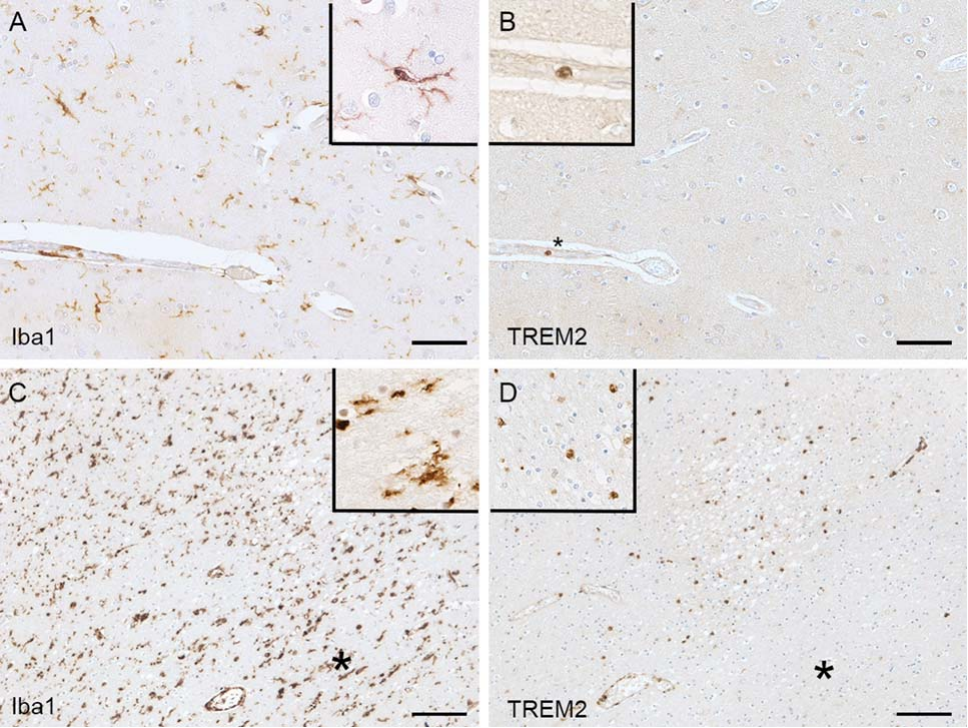
*Iba1 and TREM2 immunolabeling*. (A) Iba1 staining recognizes ramified microglia and perivascular macrophages; whereas (B) TREM2 staining in the same area detects only intravascular monocytes (*) but not microglia or perivascular macrophages (uninfarcted area). Acute infarct showing (C) high expression Iba1 in microglia and perivascular macrophages and (D) TREM2‐positive parenchymal cells within the infarct. The surrounding uninfarcted area contains Iba1‐positive (C) TREM2‐negative microglia (D)(*). The extravascular TREM2‐positive cells in the infarcted parenchyma have similar morphology to the TREM2‐positive intravascular monocytes and likely represent recruitment of circulating monocytes to the infarcted tissue. Hematoxylin counterstaining, scale bar (A, B) = 50 μm, (C, D) = 100 μm.

In the cerebral cortex, Iba1 immunohistochemistry demonstrated microglia as expected with typical morphology, having numerous short processes (ramified microglia), and perivascular macrophages (Figure 2A). In contrast, the microglia and perivascular macrophages were TREM2 negative (Figure 2B), with TREM2 immunoreactivity detected only in cells in blood vessel lumens, having round nuclei and scanty cytoplasm morphologically consistent with circulating monocytes (Figure 2B) and acting, in effect, as an internal positive control. The TREM2‐positive intravascular monocytes were observed in all 299 cases. In addition 15 of the 299 cases, in which small infarcts were detected within the sections, presented another pattern of staining as described below.

### Parenchymal TREM2 positive cells in acute infarction

Interestingly, in contrast to the situation in relatively normal brain tissue, extravascular TREM2‐positive cells were identified in regions of acute infarction included in the tissue sections. Iba1 immunohistochemistry identified microglia with mainly ramified morphology (Figure 2C), which were TREM2 negative (Figure 2D), and this was observed within the 299 cases. However, TREM2 immunolabeling was detected in extravascular parenchymal cells within the acutely infarcted zone. These TREM2 positive parenchymal cells had similar morphology to the intravascular monocytes and are likely to represent circulating monocytes that have been recruited from the circulation into the brain parenchyma in response to the infarct (Figure 2C,D).

**Figure 3:**
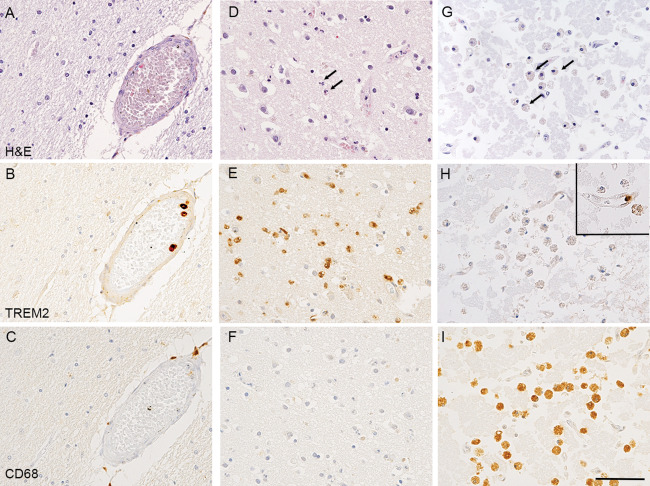
*TREM2 and CD68 immunostaining in infarcts at different stages of evolution*. (A–C) Uninfarcted region. (A) H&E staining showing preserved tissue. (B) TREM2 immunohistochemistry labelling of monocytes within the lumen of a blood vessel, but not microglia in the surrounding brain parenchyma. (C) CD68 immunohistochemistry showing staining of perivascular macrophages. (D–F) Acute cortical infarct with early neuropil disruption, neuronal ischemia and infiltration by neutrophils as observed on (D) H&E staining. (E) TREM2 immunohistochemistry showing numerous TREM2 labelled cells infiltrating the acutely infarcted cortex and present within vascular lumens. The cells have the morphology of monocytes rather than foamy macrophages or process‐bearing microglia. (F) CD68 immunohistochemistry showing no significant labelling of cells indicating the infarct is so acute that no phagocytosis of cell debris by macrophage/microglia has begun. (G) H&E staining showing an old cortical infarct with extensive neuropil disintegration and abundant foamy macrophages. (H) TREM2 immunohistochemistry showing the foamy macrophages are not labelled. A single monocyte is labelled within the lumen of a capillary within the infarct (inset top right). (I) CD68 immunohistochemistry showing immunoreactivity of abundant foamy macrophages. Counterstaining with Hematoxylin and eosin (H&E) for (A, D, G) and with Hematoxylin for (B, C, E, F, H, I); scale bar = 50 μm.

Sixteen infarcts were identified in H&E‐stained sections on morphological grounds in 15 of the 299 cases studied in total (Table 3). The histological stage of evolution of the infarcts was assessed which, in turn, could give approximate ages to the lesions (Table 2) 7, 16, 22. No parenchymal TREM2 immunoreactivity was observed in the 2 cases with very acute infarcts (<24 h) preceding a cellular reaction (Figure 3A–C), or in the six old infarct cases (>several weeks old) despite abundance of foamy macrophages and CD68 immunoreactivity at that stage (Figure 3G–I, Table 3). In contrast, TREM2 immunoreactivity in the brain parenchyma was identified in five out of 6 acute infarcts (1–3 days) (Figure 3D–F, Table 3). The TREM2‐positive cells in the acute infarcts had round nuclei and scanty cytoplasm, and were confined to the infarcted tissue, thus likely representing monocytes migrated from the circulation into the infarct. One out of 2 cases with a subacute infarct (∼1 week old, overlap stage between acute and old infarct) had both TREM2 and CD68 immunolabeled macrophages (Table 3).

**Table 3:** Expression of TREM2 and CD68 in cerebral infarcts of different ages.

ID	Infarct age	Morphological description	CD68	TREM2
1	Very acute	Very acute focal cortical ischemia (<24 h)	−	−
2	Very acute	Very acute focal cortical ischemia (<24 h)	−	−
3	Acute	Acute cortical infarct (1–3 days)	−	+++
4	Acute	Multiple acute cortical/white matter septic microemboli ± acute infarction (1–3 days)	±	+++
5	Acute	Acute lesions [Multiple cortical/white matter septic microemboli + acute (1–3 days) and subacute (i.e., at least several days) infarcts]	±	++
6	Acute	Acute cortical infarct (1–3 days)	−	+
7	Acute	Acute cortical infarct (1–3 days)	−	+
8	Acute	Acute cortical infarct (1–3 days)	−	−
9	Subacute	Subacute cortical infarct (about 1 week)	++	+++
5	Subacute	Subacute lesions [Multiple cortical/white matter septic microemboli + acute (1–3 days) and subacute (i.e., at least several days)]	++	−
10	Old	Old cortical micro‐infarct (>several months)	+	−
11	Old	Old infarct (a few weeks)	+++	−
12	Old	Old cortical infarct (many months‐years)	+	−
13	Old	Old cortical infarct (many months‐years)	+	−
14	Old	Old white matter infarct (several months)	++	−
15	Old	Old cortical micro‐infarct	++	−
16	n/a	No lesion	−	−

## Discussion

Our findings of a positive TREM2 signal in the splenic macrophages with the HPA010917 antibody are consistent with the antibody characterization study which identified that this antibody is specific for TREM2 both *in vitro* and *in vivo* 34 and expressed on myeloid cells. Our unexpected findings in the human brain revealed firstly that, although abundant microglia were identified by immunoreactivity for Iba1, the microglia did not express TREM2 in the 299 cases of the CFAS cohort. This was despite TREM2 positivity of monocytes within the lumens of blood vessels in the sections of brain tissue, representing monocytes circulating in the blood. Secondly, TREM2‐positive cells were present within the brain parenchyma in acute infarcts (1–3 days approximately). These TREM2‐positive cells had the morphology of monocytes and macrophages, rather than process‐bearing microglia, and seem likely to be monocytes recruited from the bloodstream in response to the infarct; subsequently evolving into macrophages as they begin to phagocytose the necrotic tissue. As expected, these acute infarcts prior to the onset of phagocytosis, lacked staining with the lysosomal marker CD68. In contrast, older infarcts in which phagocytosis is taking place contained CD68‐positive macrophages, consistent with previous studies in humans and laboratory animals 1, 9, 29, but these macrophages lacked TREM2 immunoreactivity. Interestingly, one of the two subacute infarcts cases had immunoreactivity of cells for both TREM2 and CD68. These findings imply that following infarction and the breakdown of the blood‐brain barrier, TREM2‐positive CD68‐negative monocytes invade the brain parenchyma at the site of the injury (consistent with the role of TREM2 expression in chemotaxis 30), differentiate into TREM2‐positive CD68‐positive macrophages that became, over a period of about a week, phagocytic CD68‐positive TREM2‐negative macrophages. The subacute infarct, approximately one week in age 7, 16, 22, support this phenotypic change by having both TREM2 and CD68 immunoreactivity in morphologically macrophage‐like cells present within the damaged parenchyma. In the cases with infarcts, in the surrounding uninvolved brain parenchyma microglia were immunoreactive for Iba1, showed variable degrees of CD68 expression, but were consistently unlabelled for TREM2 31.

Overall, it appears that the TREM2 antibody employed in this study (HPA010917) labels specifically monocytes circulating in the bloodstream and monocytes recruited from the blood into the brain parenchyma in response to tissue injury, but not microglia resident within the brain. We are acknowledging that the level of TREM2 expression in microglia could be below the level of detection by our methods. It is theoretically possible that lack of microglial immunoreactivity with this antibody could be due to cleavage of the TREM2 protein proximal to the binding site of the antibody, but such cleavage would have to be selective for microglia and not monocytes or macrophages. The antibody has been raised against a recombinant 35 amino acid sequence (amino acids 196–230) of TREM2 corresponding to the cytoplasmic sequence. The identified cleavage site of TREM2 C‐terminal to histidine 157 sheds an ectodomain but appears to leave the HPA010917 antibody binding site within the cell cytoplasm 24, 40, consistent with the cytoplasmic signal detected on the tissue.

Our observations are consistent with: (i) the original study of the characterization of TREM2 in human immature monocyte‐derived dendritic cells 2, (ii) the findings in one control and one AD case using the same antibody in the previous study assessing the sensitivity of TREM2 antibodies by Western blot followed for the HPA010917 antibody by immunohistochemistry after preabsorption with the recombinant protein to confirm the specificity 34, (iii) with the detection of TREM2 expression in in monocyte‐derived macrophages but not microglia in APP/PS1 mice 19, and (iv) the absence of association between TREM2 and Iba1 protein levels detected by Western blots in human frontal cortex 33. Our findings are also in accord with the proposed role of TREM2 as regulator of phagocytosis, as the brain parenchymal cells labelled in this study are indeed performing a phagocytic function in clearing necrotic tissue debris in infarcts.

We did not observe any TREM2 staining around plaques in the CFAS cases with AD pathology, therefore providing no evidence that in humans peri‐plaque microglia are derived from circulating monocytes. Our observation appears different from a previous study on 11 *post‐mortem* cases that describes TREM2 immunoreactivity on microglia associated with plaques using the R&D AF1828 antibody. In this study, the authors checked the specificity of the antibody using Western blot analysis but they did not carry out TREM2 immunodetection on a positive myeloid tissue 28. A previous study demonstrated that whereas this antibody was able to recognize human recombinant TREM2 on Western blot, it was not able to label monocytes/macrophages, dendritic cells or osteoclasts on human tissue 34. Of note, the authors did not perform single immunodetection of TREM2, but instead presented high magnification pictures of double staining with HLA‐DR using light microscope. The quality of the staining required arrows to indicate the positive cells questioning its reliability.

Our findings counter the observations in animal models, such as the ones in the APP23 and the *APPswe/PS1dE9* models using a Santa Cruz TREM2 antibody 8, 20 or in the 5XFAD using a R&D TREM2 antibody 45. These studies observed (i) upregulation of TREM2 expression during disease progression, (ii) TREM2‐positive microglia, and (iii) TREM2‐positive microglia around plaques. Therefore, our own observations appear to challenge the fidelity of the animal models relating to this aspect of the complexity of human AD pathophysiology. Importantly, the time‐frame also differs: in the experimental models, the response was typically studied after a matter of few months; whereas the brains of the patients with dementia are being studied several years following the onset of the disease. However, one of the strengths of the CFAS cohort is that it is the result of an unbiased representation of the elderly population and thus includes people at all stages of AD including early stages of plaque formation. Some human studies have identified the presence of TREM2 in human brain tissue using biochemical methods only (e.g., Western blot, mRNA). They do not contradict our findings as human brain tissue homogenates will include circulating monocytes within the lumens of blood vessels and so these methods will not distinguish between these cells and microglia.

Our study in a large cohort of human *post‐mortem* cases does not contradict the evidence for the role of TREM2 and the immune system as risk factors for AD despite the absence of TREM2‐positive microglia. The genetic data suggest that *TREM2* gene variation, although rare, can alter risk as substantially as APOE genotype 10, 21. Instead, it draws attention to an important role for peripheral myeloid cells, consistent with a role for systemic inflammation, in the development and progression of AD 13. Of note, a recent study observed increased peripheral blood *TREM2* mRNA in AD is associated with cognitive decline and hippocampal atrophy, supporting TREM2 as a putative peripheral biomarker for AD 39.

Several pieces of evidence are in favour of a communication between the systemic immune system and the brain. Experimental studies have demonstrated that systemic inflammation whether in the form of a chronic disease (e.g., osteoarthritis), or infection (e.g., LPS) can enhance AD pathology in animal models 23, 26, 27, 37. This led to the prediction that systemic infection and inflammation could exaggerate the acute symptoms of disease, increase the ongoing tissue injury and impact on the rate of disease progression. In clinical studies, the presence of systemic infection and inflammation, with raised peripheral pro‐inflammatory cytokines, was associated with a marked increase in the rate of long‐term cognitive decline and the neuropsychiatric features typical of sickness behavior in AD patients 14, 15, 18. This finding is consistent with information from *in vivo* PET imaging showing that the cognitive decline is associated with the inflammatory signal in the brain 6. The use of the tumor necrosis factor (TNF)‐α inhibitor etanercept in a small randomized, placebo‐controlled, double‐blind study to block low‐grade peripheral systemic inflammation in AD patients showed trends that favor etanercept compared to placebo 4.

Further studies with additional antibodies to TREM2, as they are developed, will help clarify our understanding of this protein which genetics indicates is important in AD pathogenesis. Overall, our demonstration here that despite *TREM2* gene variation influencing risk of AD, TREM2 positive cells are largely restricted to the blood circulation, promotes a role for systemic inflammation in the development and progression of AD, and highlights the need for a greater understanding of the communication involved between the periphery and the brain.

## Conflict of Interest

The authors do not have conflict of interest.

## Ethics

The study received ethical approval from the Cambridgeshire 1 Research Ethics Committee (Rec number: 10/H0304/61/).

## Contributors

MF performed the experiments and collected the data; SR tested the specificity of the antibodies; JC prepared the sections of the 299 cases; CB provided the clinical information; PGI provided the neuropathological data; JARN reviewed and classified the infarct cases and with DB conceived the study; DB wrote the manuscript.
